# Prefoldin subunit 6 of *Plasmodium falciparum* binds merozoite surface protein‐1

**DOI:** 10.1002/2211-5463.13022

**Published:** 2022-03-29

**Authors:** Vikash Kumar, Rumaisha Shoaib, Ankita Behl, Akshay Munjal, Mohammad Abid, Shailja Singh

**Affiliations:** ^1^ Special Centre for Molecular Medicine Jawaharlal Nehru University New Delhi India; ^2^ Medicinal Chemistry Laboratory Department of Biosciences Faculty of Natural Sciences Jamia Millia Islamia New Delhi India

**Keywords:** chaperone, malaria, merozoite surface protein‐1, *Plasmodium falciparum*, prefoldin

## Abstract

Malaria is a human disease caused by eukaryotic protozoan parasites of the *Plasmodium* genus. *Plasmodium falciparum* (*Pf*) causes the most lethal form of human malaria and is responsible for widespread mortality worldwide. Prefoldin is a heterohexameric molecular complex that binds and delivers unfolded proteins to chaperonin for correct folding. The prefoldin PFD6 is predicted to interact with merozoite surface protein‐1 (MSP‐1), a protein well known to play a pivotal role in erythrocyte binding and invasion by *Plasmodium* merozoites. We previously found that the *P. falciparum* (*Pf*) genome contains six prefoldin genes and a prefoldin‐like gene whose molecular functions are unidentified. Here, we analyzed the expression of *Pf*PFD‐6 during the asexual blood stages of the parasite and investigated its interacting partners. *Pf*PFD‐6 was found to be significantly expressed at the trophozoite and schizont stages. Pull‐down assays suggest *Pf*PFD‐6 interacts with MSP‐1. *In silico* analysis suggested critical residues involved in the *Pf*PFD‐6‐MSP‐1 interaction. Our data suggest *Pf*PFD‐6 may play a role in stabilizing or trafficking MSP‐1.

## Graphical abstract

Prefoldin is a cochaperone that delivers unfolded proteins to chaperonin for proper folding. Here, we have characterized prefoldin‐6 subunit (PfPFD‐6) of *Plasmodium falciparum*. PfPFD‐6 is expressed during asexual blood stages and interacts with merozoite surface protein‐1 (MSP‐1), a key protein involved in erythrocyte invasion by the malaria parasite. Our study suggests that PfPFD‐6 may play a role in stabilizing or trafficking MSP‐1.

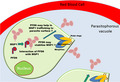

**Abbreviations**
GST: glutathione *S*‐transferase
IFA: immunofluorescence assay
MSP‐1: merozoite surface protein‐1
Ni‐NTA: nickel–nitrilotriacetic acid
*Pf*
: *Plasmodium falciparum*
PFD: prefoldin
RBCs: red blood cells
RT–PCR: reverse transcription polymerase chain reaction

Malaria is a major human health concern caused by the eukaryotic protozoan parasite ‘*Plasmodium*’. Among *Plasmodium* species, *Plasmodium falciparum* (*Pf*) causes the most lethal form of human malaria and is responsible for widespread mortality worldwide [1]. Humans become infected when a female *Anopheles* mosquito transfers parasite through its saliva, which migrate through the skin into the host blood stream [2, 3]. The parasite then infects the hepatocytes where it undergoes asexual multiplication. This is followed by the release of merozoites, which specifically invade the host erythrocytes to commence asexual life cycle [4]. This specificity is associated with receptor–ligand‐type interactions between merozoites and erythrocytes [5, 6]. It has been believed that initial interactions are mediated by merozoite surface proteins (MSPs) through multiple weak interactions with receptors on the surface of RBCs [7, 8]. Among MSPs, merozoite surface protein‐1 (MSP‐1) is best characterized and reports suggest its role in erythrocyte invasion [8, 9, 10]. Uniform distribution of MSP‐1 over the merozoite surface and the observation that antibodies against MSP‐1 inhibit invasion have implicated its role in host cell invasion [10]. Another interesting aspect about MSP‐1 is that it is essential for parasite viability [11] and undergoes proteolytic processing that is coincident with merozoite maturation and invasion [12, 13, 14, 15, 16].

Molecular chaperones are ubiquitous proteins, which play key roles in protein folding, trafficking, and degradation of proteins within the cell, and are critical for maintaining cellular homeostasis [17, 18, 19]. Among chaperones, ATP‐dependent group of proteins known as chaperonin are characterized by double‐ring structure that are found in both prokaryotes and eukaryotes [20, 21, 22, 23]. Based on the presence or absence of a co‐chaperonin, chaperonins are classified into two groups, that is, group I and group II chaperonins [24]. Group I chaperonins expressed in bacterial cytoplasm (GroEL) and endosymbiotic organelles [25], whereas group II chaperonins are found in archae (thermosome) and in the eukaryotic cytosol as TCP‐1 ring complex (TRiC or CCT) [26]. Both chaperonins share common structures with different functions [27].

Prefoldin (PFD) is widely regarded as a cochaperone of group II chaperonin in eukaryotes and was first recognized as Gim (genes involved in microtubule biogenesis) in yeast [23, 28]. PFD plays a central role in stabilizing unfolded proteins and subsequently deliver them to group II chaperonin to facilitate correct folding [23, 28, 29, 30]. Archaeal PFDs are comprised of two types of subunits (two α subunits and four β subunits), whereas eukaryotic PFDs are composed of six different subunits (two α‐like subunits: PFD3 and PFD5 and four β‐like subunits: PFD1, PFD2, PFD4, and PFD6) [31]. Archaeal PFDs have been shown to stabilize nascent proteins and prevent them from aggregation [32, 33]. In eukaryotes, PFDs mainly bind to nascent cytoskeletal proteins and protect them from unwanted interactions [31, 34, 35].

Prefoldins have been reported to play key roles in several necessary cellular processes. In *Caenorhabditis  elegans*, reduction in functional PFDs by RNAi leads to defects in cell division that ultimately causes embryonic lethality [36]. Deletion of single or multiple subunits of PFD genes causes cytoskeletal defects, slow growth, and low temperature stress in yeast [37, 38, 39]. In *Arabidopsis*, PFD6 mutant causes a range of deformability that includes defect in microtubule and cell division, cortical array organization, and microtubule dynamicity [40, 41, 42]. Recent studies show that eukaryotic PFD plays an important role in quality control against protein aggregation. Moreover, dysfunction of PFD leads to neurodegenerative diseases [43, 44]. Another reports suggest that PFD1 promotes epithelial–mesenchymal transition (EMT) and lung cancer progression by suppressing expression of cyclin A by binding to its promoter at transcription start site [45, 46].

Although the role of PFDs has been described in archae and eukaryotes, their functions in *P. falciparum* remain largely unidentified. Lilburn *et al*. studied the heat‐shock response network of *P. falciparum* by integrating available high‐throughput omics data. They found putative prefoldin subunits namely prefoldin 6 (PF3D7_051200), prefoldin 3 (PF3D7_071850), prefoldin 4 (PF3D7_090450), cochaperone prefoldin complex subunit 5 (PF3D7_112810), and prefoldin 2 (PF3D7_1416900) as heat‐shock proteins that had protein–protein associations. This interactome of heat‐shock response‐related proteins is believed to a play crucial role in the survival of parasite during febrile episodes of temperature fluctuations [47]. A more recent study showed upregulated expression of prefoldin ‘FAZP’ in artesunate (ART)‐resistant line of *Leishmania donovani*. FAZP is associated with ART resistance in *P. falciparum* malaria [48].

We searched in Plasmodb database using the keyword ‘prefoldin’ and found that *P. falciparum* encodes six PFD subunits and a prefoldin‐like protein [49]. According to yeast two‐hybrid (Y2H) data available on PlasmoDb, PFD6 is found to interact with merozoite surface protein‐1 (MSP‐1), a protein well known to play a pivotal role in erythrocyte binding and invasion by *Plasmodium* merozoites. This looks intriguing that how a small prefoldin subunit of molecular mass 13 kDa associates and stabilizes a large protein (MSP1) of mass ~ 200 kDa, which is indispensable for invasion of the red cell by the parasite. This led us to look closer into PFD6 among all prefoldin subunits. In this study, we have attempted to delineate the function of *Pf*PFD‐6 (Plasmodb Id: PF3D7_0512000; [49]) using pull‐down assays and confirmed its expression in *Plasmodium* asexual blood stages by RT–PCR, immunofluorescence assay (IFA), and western blotting. *Pf*PFD‐6 was found to interact with MSP‐1, which shed light on the probable role of *Pf*PFD‐6 in MSP‐1 stability and indirectly in erythrocyte invasion.

## Materials and methods

### Parasite culture

*Plasmodium falciparum* 3D7 parasites were cultured in O+ RBCs using complete RPMI 1640 medium supplemented with 0.5 g·L^−1^ AlbuMAX I (Gibco, Dún Laoghaire, Dublin, Ireland), 27.2 mg·L^−1^ hypoxanthine (Sigma, St. Louis, Missouri, USA), and 2 g·L^−1^ sodium bicarbonate (Sigma). Culture was maintained at 37 °C in 90% N_2_, 5% CO_2_, and 5% O_2_ containing environment and maintained at 5% hematocrit and 5% parasitemia. Late stages of schizonts were harvested by centrifugation from *Plasmodium* cultures (parasitemia 8–10%), and the parasites were released from red blood cells by treatment with 0.15% saponin. Parasite pellet was washed with 1× PBS and stored at −80 °C for experiments. Parasites were synchronized by 5% sorbitol (Sigma) selection of rings, and late trophozoites or schizonts were purified from mixed parasite culture using 65% Percoll (Sigma).

### Cloning, expression, purification, and antibody generation of recombinant *Pf*PFDN‐6

Gene encoding full‐length PF3D7_0512000 (360 bp size; *Pf*PFD‐6) was PCR‐amplified from cDNA using gene‐specific forward (5′ ATGTCTCAAGAAAAAATTAGTGAA 3′) and reverse (5′ TTAAGCTTGTGGAACGGGTAT 3′) primers. PCR‐amplified product was cloned into pGEX4T‐1 and pET28a vector. Full constructs were expressed as C‐terminal hexahistidine‐tagged and GST‐tagged fusion protein in BL21 (DE3) *Escherichia coli* cells. Affinity purification was carried out for His‐tagged recombinant protein in buffer containing 50 mm Tris/HCl, 300 mm NaCl, and 0.02% Na/azide, pH 8.0. Elutes of affinity chromatography of *Pf*PFD‐6 were subjected to anion exchange chromatography using a Q Sepharose column (GE Healthcare Life Sciences, Chicago, IL, USA). GST‐tagged recombinant protein was purified in 50 mm Tris, 150 mm NaCl, and 0.02% Na/azide pH 8.0 by using glutathione affinity chromatography.

### RNA isolation and RT–PCR of *Pf*PFD‐6 gene

RNA was isolated from all the three stages of parasite (16–20 h of ring, 32–38 h of trophozoite, and 40–46 h of schizont) using TRIzol reagent (Life Technologies, Carlsbad, CA, USA) following standard protocol [50]. DNA was removed by the DNase treatment kit (DNA‐*free*™ DNA Removal Kit; Invitrogen, Thermo Fisher Scientific, Waltham, MA, USA). Purity and concentration of RNA were estimated. Two microgram of total RNA was reverse‐transcribed into cDNA using the cDNA Kit (Thermo Fisher scientific) according to the manufacturer's protocol. One hundred nanogram of cDNA from each stage was used for semi‐quantitative PCR by using specific *Pf*PFD‐6 primers. 18s primer was used as a loading control.

### Raising polyclonal antisera and *in vivo* expression analysis of *Pf*PFD‐6

Polyclonal antibodies were raised in house in male New Zealand White rabbits and male BALB/c mice using purified recombinant proteins as immunogens following standard protocol [51]. Briefly, animals were immunized with emulsion containing 1 : 1 ratio of Freund's complete adjuvant and antigen followed by three booster doses at an interval of 14 days. Booster doses comprise 1 : 1 ratio of Freund's incomplete adjuvant and antigen. Final bleed was collected, and the end point titers of raised antisera were determined by western blotting.

For *in vivo* expression analysis, *Pf*3D7 schizont stage asexual cultures (parasitemia ~ 8%) were subjected to saponin lysis (0.15% w/v) followed by extensive washing of parasite pellet with 1× PBS to remove traces of hemoglobin. Parasite pellet (~ 10 µg total protein) was resolved on 12% SDS/PAGE and transferred to nitrocellulose membrane. The blot was blocked in 5% BSA in 1× PBS overnight at 4 °C and probed with rabbit anti‐*Pf*PFD‐6 antisera (1 : 5000) followed by incubation with horseradish peroxidase (HRP)‐conjugated goat anti‐rabbit IgG (1 : 5000; Sigma‐Aldrich, St. Louis, MO, USA). The blot was developed using Enhanced Chemiluminescence Kit (Bio‐Rad, Hercules, CA, USA).

### Colocalization assay

Thin blood smears of mixed stage *Pf*3D7 cultures at 5% parasitemia were fixed in methanol for 45 min at −20 °C, permeabilized with 0.05% PBS/Tween 20, and blocked with 5% (w/v) BSA in PBS. For colocalization studies, mouse anti‐*Pf*PFD‐6 (1 : 250) and rabbit anti‐MSP‐1 (1 : 250) [52] were added as primary antibodies and incubated for 2 h at room temperature. Alexa Fluor 488‐conjugated anti‐rabbit (1 : 500, red color; Molecular Probes, Invitrogen, Carlsbad, CA, USA) and Alexa Fluor 546‐conjugated anti‐mouse (1 : 500, green color; Molecular Probes) were used as secondary antibodies. The parasite nuclei were counterstained with DAPI (40, 60‐diamidino‐2‐phenylindole; Invitrogen) and mounted with a coverslip. The slides were examined using a confocal microscope (Olympus, Shinjuku, Tokyo, Japan) with a 9 100 oil‐immersion objective.

### Pull‐down assay

Pull‐down assays were performed to test *Pf*PFD‐6 and MSP‐1 interaction. Briefly, *E. coli* BL21 (DE3) cells containing pGEX4T‐1 with and without *Pf*PFD‐6 insert were grown and harvested. Culture was lysed, and bacterial lysate was incubated with Glutathione Sepharose beads for binding. Beads bound with GST‐fused *Pf*PFD‐6 and only GST‐bound beads were incubated with parasite lysate comprising majorly of trophozoites and late schizonts for 3 h at 4 °C. The beads were washed extensively three times with the washing buffer (20 mm HEPES, 150 mm NaCl, pH 7.4). The complex was recovered from the beads by eluting in elution buffer containing reduced 20 mm glutathione in 50 mm Tris/Cl. Eluted fraction and protein complex bound beads were resolved on 12% SDS/PAGE, transferred to nitrocellulose membrane, and probed with monoclonal anti‐MSP1_19_ (1 : 500) followed by incubation with horseradish peroxidase (HRP)‐conjugated goat anti‐rabbit IgG (1 : 5000; Sigma‐Aldrich). Eluted fraction and protein complex bound beads were also probed with anti‐*Pf*PFD‐6 and anti‐GST antibodies on the same blot.

### Homology modeling and docking of *Pf*PFD‐6 and MSP‐1

Three‐dimensional structures of *Pf*PFD‐6 and MSP‐1 (amino acids 241–1700) were constructed by I‐TASSER server [53]. The built models were subjected to refinement and energy minimization using 3D refine and Swiss‐PdbViewer (SPDBV), respectively [54, 55]. Procheck and ERRAT servers were used to check the stereochemical quality and overall energy of predicted model, respectively [56, 57].

For docking studies, *Pf*PFD‐6 was docked with MSP‐1 model using HDOCK web server. The RIN profile of obtained representative structure of *Pf*PFD‐6 and MSP‐1 complex was generated using RING 2.0 web server [58]. RIN analysis is a technique that represents various interactions in the form of a detailed network model.

## Results

### Cloning, expression, and purification of *Pf*PFD‐6 protein

**Fig. 1:**
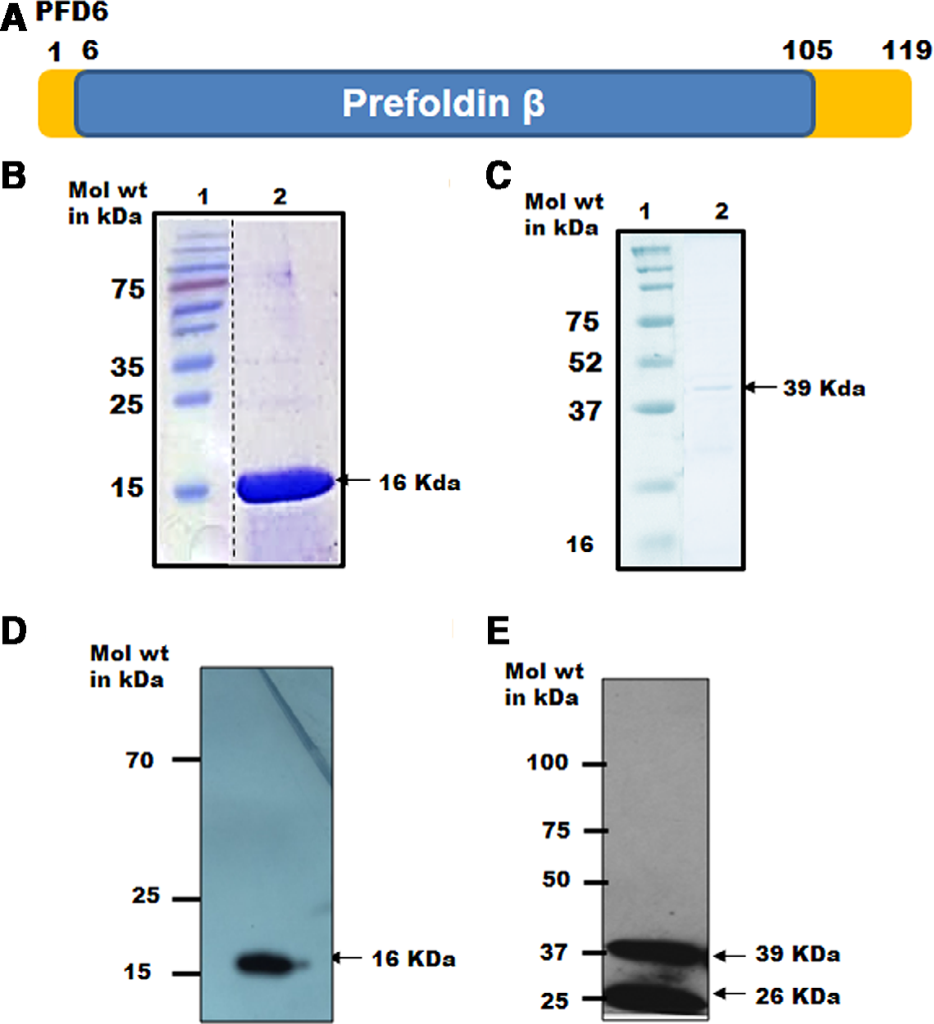
Domain organization and expression of *Pf*PFD‐6 protein. (A) Domain organization of PF3D7_0512000 (*Pf*PFD‐6). Schematic representation of full‐length *Pf*PFD‐6 protein (yellow) containing a prefoldin β‐superfamily domain (blue) (B) SDS/PAGE of purified recombinant protein cloned in pET28a vector lane 1: protein ladder; lane 2: purified recombinant *Pf*PFD‐6. Dashed line indicates that lanes were not originally adjacent. (C) SDS/PAGE of purified recombinant protein cloned in pGEX4T‐1 vector. Lane 1: protein ladder; lane 2: purified recombinant *Pf*PFD‐6 (D) Western blot of purified recombinant protein using antihistidine antibodies. (E) Western blot of purified recombinant protein using anti‐GST antibodies.

**Fig. 2:**
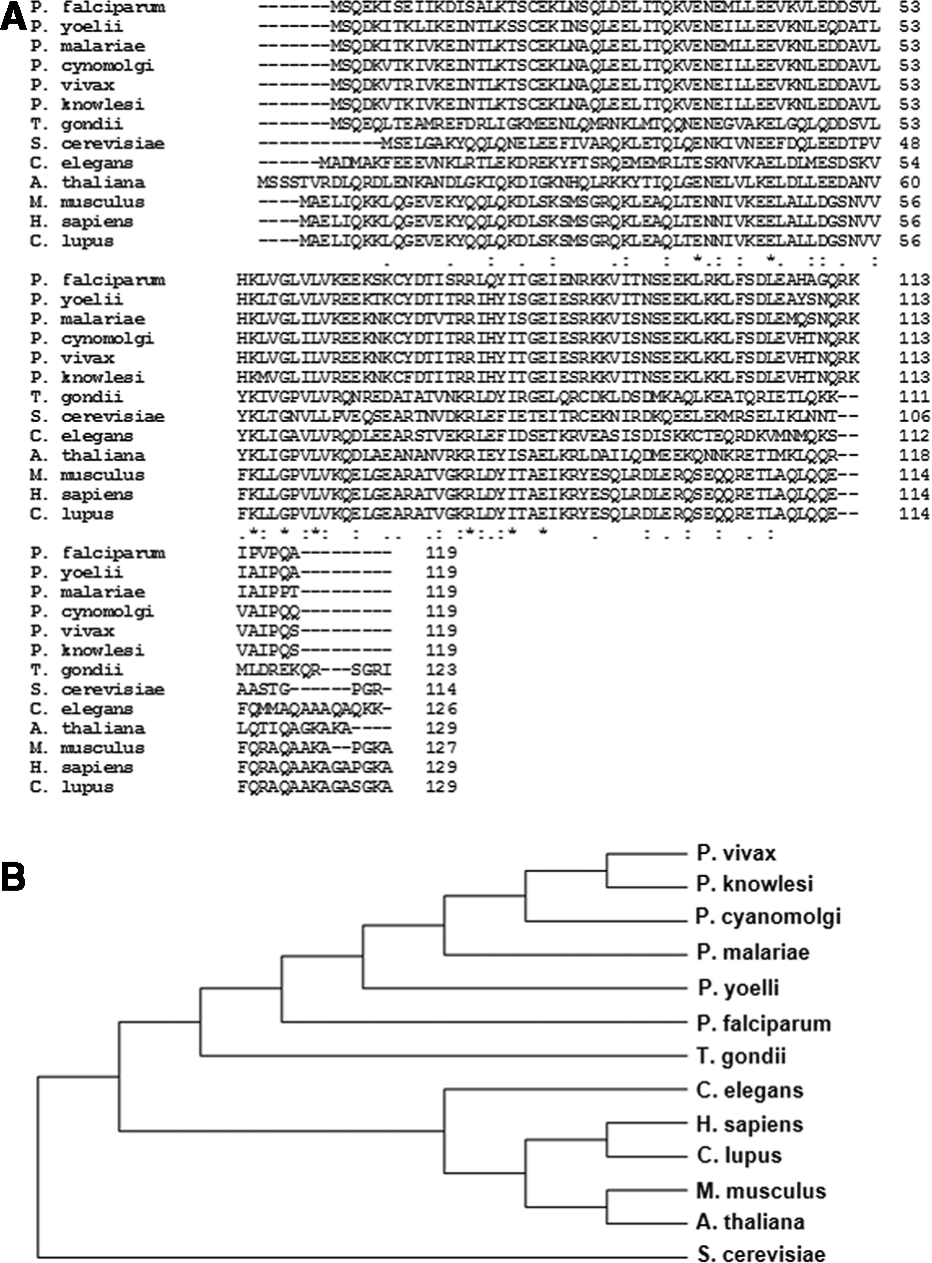
Multiple sequence alignment and phylogenetic tree of *Pf*PFD‐6 subunits with its homologs. (A) Comparison of amino acid sequence of *Pf*PFD‐6 subunits with its homologs in *Plasmodium* species, *Homo sapiens*, *T. gondii*, *C. elegans*, *Saccharomyces cerevisiae*, *A. thaliana*, *M. musculus*, and *C. lupus*. Alignment was performed using clustal omega, EMBL‐EBI, Hinxton, Cambridge, UK. ‘*’ represents conserved residues, whereas ‘:’ and ‘.’ represent semi‐conserved residues. (B) Phylogenetic tree of *Pf*PFD‐6 subunits with its homologs using mega 6 software, Pennsylvania State University, PA, USA.

The *Pf*PFD‐6 gene was cloned in pET‐28a (+) and pGEX‐4 T1 vector (Novagen, Vadodara GJ, India) and expressed in *E. coli* BL21 (DE3) cells. Figure 1A shows a schematic representation of full‐length *Pf*PFD‐6 protein (119 amino acid residues; yellow color) harboring a prefoldin β‐domain (100 amino acid residues; blue color). Expression of N‐terminal hexahistidine (His)‐tagged and C‐terminal GST‐tagged *Pf*PFD‐6 constructs was scaled up to purify protein from the soluble fraction using various chromatographic techniques. Recombinant GST‐tagged *Pf*PFD‐6 was purified by affinity chromatography using GST beads, whereas His‐tagged *Pf*PFD‐6 was purified using nickel/nitrilotriacetic acid (Ni‐NTA) resin. His‐tagged protein was further purified by ion‐exchange chromatography. Purified recombinant His‐tagged *Pf*PFD‐6 and GST‐tagged *Pf*PFD‐6 run as a species of ~ 16 and ~ 39 kDa, respectively, on SDS/PAGE (Fig. 1B,C). Identity of His‐tagged *Pf*PFD‐6 and GST‐tagged *Pf*PFD‐6 was confirmed by western blotting using anti‐His monoclonal and anti‐GST antibodies, respectively (Fig. 1D,E). Multiple sequence alignment of *Pf*PFD‐6 with its homologs in *Plasmodium* species, *Homo sapiens*, *Toxoplasma gondii*, *  Caenorhabditis elegans*, *Saccharomyces cerevisiae*, *Arabidopsis thaliana*, * Mus musculus*, and *Canis lupus* showed significant conservation among PFD6 proteins (Fig. 2A). Phylogenetic tree depicting the sequence relationship among these PFD6 proteins is represented in Fig. 2B.

### Expression profile in the intra‐erythrocytic cycle by RT–PCR

Stage‐specific real‐time PCR assays on 18S rRNA were performed to investigate the expression of *Pf*PFD‐6 gene during asexual blood stages (i.e., rings, trophozoites, and schizonts) of parasite life cycle. Parasites were synchronized, and RNA was extracted from three intra‐erythrocytic asexual blood stages of the parasite. Our result revealed that the expression of *Pf*PFD‐6 transcripts at all asexual parasite stages. 18S rRNA was used as a loading control to clarify equal loading of cDNA sample (Fig. S1A).

### Confirmation of *in vivo* expression of *Pf*PFD‐6

**Fig. 3:**
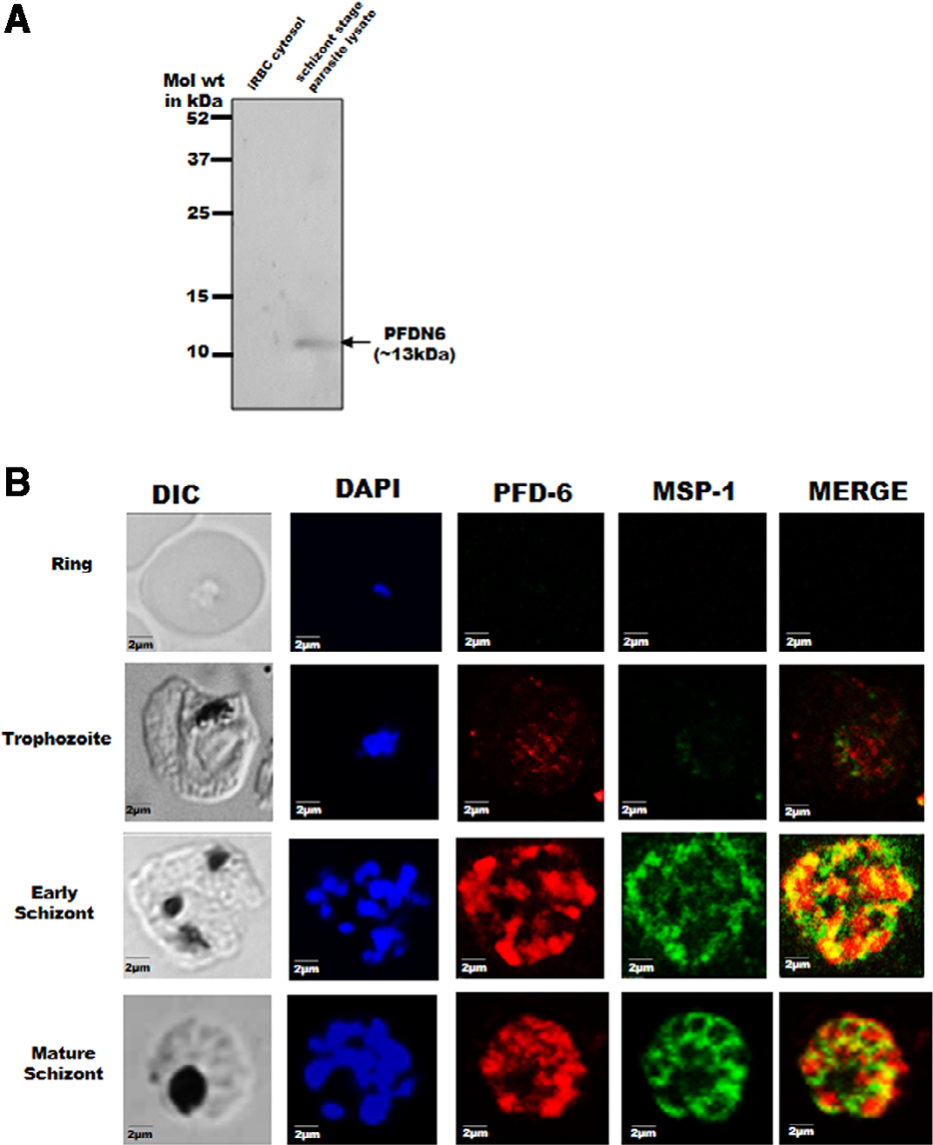
*In vivo* expression and colocalization assay. (A) Western blot analysis of schizont stage *Pf*3D7 parasite lysates to test *in vivo* expression of *Pf*PFD‐6. Lane 1: protein ladder; lane 2: schizont stage parasite lysate. Blot was probed with anti‐*Pf*PFD‐6 antibodies followed by secondary antibodies. (B) Expression analysis and colocalization of *Pf*PFD‐6 with MSP‐1 at different asexual stages of parasite life cycle. Smears of methanol‐fixed *Pf*3D7‐infected erythrocytes were stained with anti‐*Pf*PFD‐6 antibodies (1 : 250) and anti‐MSP‐1 antibodies (1 : 250), followed by incubation with Alexa Fluor‐conjugated secondary antibodies (Alexa Fluor 488, red; Alexa Fluor 546, green). DIC, differential interference contrast image; DAPI, nuclear staining 40, 6‐diamidino‐2‐phenylindole (blue); MSP‐1, mouse anti‐MSP‐1 (green); *Pf*PFD‐6, anti‐*Pf*PFD‐6 antibody (red); merge, overlay of *Pf*PFD‐6 proteins with MSP‐1. Scale bar represents 2 μm.

Polyclonal antibodies against *Pf*PFD‐6 were raised in house in rabbits and mice using purified recombinant *Pf*PFD‐6 and tested for reactivity on *Pf*PFD‐6. These antibodies were checked for their specificity against *Pf*PFD‐6 prior to their use. Detection of a single band of induced *Pf*PFD‐6 in crude extract of *E. coli* BL21 (DE3) transformed with its cloned plasmid validated the specificity of these antibodies (lane 4; Fig. S1B,C). Crude extract of *E. coli* BL21 (DE3), uninfected red blood cell pellet, and uninfected red blood cell cytosol were used as a negative controls (lanes 1, 2, and 3; Fig. S1B,C). Signal was absent when samples were probed with pre‐immune sera (data not shown). Western blot analysis was then conducted on schizont stage parasite lysate and iRBC cytosol using rabbit anti‐PFD6 antisera to check *in vivo* expression of PFD protein during the asexual blood stages of *Pf*. A single band of expected molecular weight for *Pf*PFD‐6 (13 kDa) was detected only in the parasite lysate, suggesting the expression of *Pf*PFD‐6 in asexual blood stages of parasite life cycle (Fig. 3A; lane 2). No band was detected in infected red blood cells cytosol, suggesting the presence of *Pf*PFD‐6 within parasite (Fig. 3A; lane 1).

### *Pf*PFD‐6 colocalizes with MSP‐1

Yeast two‐hybrid data of *Plasmodium* PFDs showed that *Pf*PFD‐6 interact with merozoite surface protein 1 (MSP‐1) [49]. MSP‐1 is considered as a candidate for blood‐stage malaria vaccines owing to its role in host erythrocyte binding and invasion [59, 60]. Immunofluorescence assays (IFAs) were performed to visualize the expression of *Pf*PFD‐6 and colocalization of *Pf*PFD‐6 with MSP‐1 during the asexual stages of *Pf* using protein‐specific antibodies on cultured parasites (Fig. 3B). The expression of *Pf*PFD‐6 and MSP‐1 begins at mid‐trophozoite stage of parasite's life cycle. At schizont stages, these seem to remain confined within the parasitophorous vacuole (PV) of *Pf*. *Pf*PFD‐6 showed negligible colocalization with MSP‐1 at the trophozoite stage of parasite development. In the schizont stage, significant overlapping of signals was observed within the PVM, suggestive of their coexistence (Fig. 3B). At schizont stage, MSP‐1 mostly localizes to PVM of *Pf*. However, to ascertain confinement of fluorescence within the PV and PVM, costaining with PV and PVM marker proteins would be required.

### *Pf*PFD‐6 binds to merozoite surface protein‐1 of *Plasmodium falciparum*

**Fig. 4:**
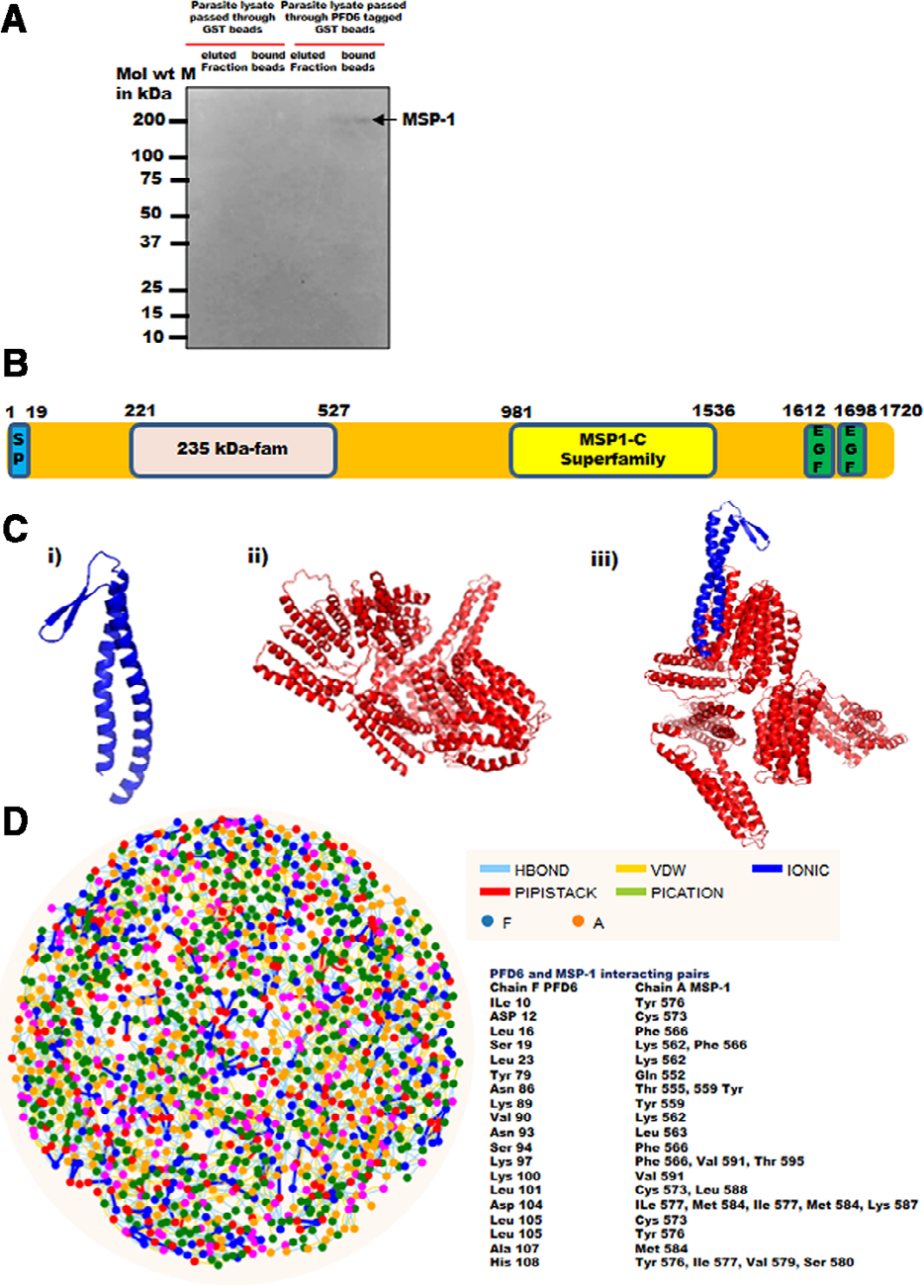
Domain organization of MSP‐1 protein and interaction of *Pf*PFD‐6 with MSP‐1. (A) Pull‐down assay of *Pf*PFD‐6 with MSP‐1. Beads bound with GST‐tagged *Pf*PFD‐6 and only GST‐bound beads were incubated with schizont stage parasite lysate. The protein complex was recovered from the beads by eluting in elution buffer. Eluted fraction and protein complex bound beads were resolved on 12% SDS/PAGE, transferred to nitrocellulose membrane, and probed with monoclonal anti‐MSP1_19_ (1 : 500). Lane M: protein ladder, lane 1: eluted fraction of GST‐bound beads, lane 2: supernatant of boiled GST‐bound beads, lane 3: eluted fraction of GST‐tagged *Pf*PFD‐6‐bound beads, and lane 4: supernatant of boiled GST‐tagged *Pf*PFD‐6‐bound beads. (B) Schematic representation of MSP‐1 showing signal peptide (SP, blue), 235 kDa fam domain (pink), MSP1‐C superfamily domain (yellow), and EGF domain (green). (C) Cartoon representation of modeled structures. (i) Model structure of *Pf*PFD‐6, (ii) model structure of MSP‐1, and (iii) docked structure of *Pf*PFD‐6‐MSP‐1 complex. (D) RIN plot of *Pf*PFD‐6‐MSP‐docked complex. Nodes in blue and orange on the plot represent residues of *Pf*PFD‐6 and MSP‐1, respectively. Pairs of interacting residues are mentioned on the plot (right panel).

After observing colocalization of *Pf*PFD‐6 with MSP‐1, we tested the binding of *Pf*PFD‐6 with MSP‐1. GST pull‐down assay was performed to analyze the ability of *Pf*PFD‐6 to pull down MSP‐1 from parasite lysate. Bacterial lysate‐containing GST‐tagged *Pf*PFD‐6 protein and only GST were coupled to Glutathione Sepharose beads followed by incubation with schizont lysates. After extensive washing, the protein fractions were eluted and remaining protein‐bound beads were resolved on SDS/PAGE followed by probing with monoclonal anti‐MSP‐1 antibodies. A ~ 200 kDa band of full‐length MSP‐1 protein was observed in lane where beads containing GST‐tagged *Pf*PFD‐6 were incubated with parasite lysate. In this lane, the supernatant of these beads was loaded after boiling (lane: 4; Fig. 4A). While no band was observed in elution fraction from only GST‐bound beads, supernatant from boiled GST‐bound beads, elution fraction of *Pf*PDF‐6‐bound beads (lanes: 1, 2, and 3; Fig. 4A). In parallel, we resolved all these fractions on SDS/PAGE followed by probing with anti‐*Pf*PFD‐6 and anti‐GST antibodies on the same blot. Our western blot data revealed a band size of 26 kDa (only GST) obtained in elution fraction from only GST‐bound beads and supernatant from boiled GST‐bound beads (lanes 1 and 2; Fig. S1D). Also, a 26 kDa (only GST) band appeared in elution fraction of *Pf*PDF‐6‐bound beads (lane 3; Fig. S1D) and two bands corresponding to molecular sizes of 39 kDa (PFD6 + GST) and 26 kDa (only GST) appeared in supernatant of boiled GST‐tagged *Pf*PFD‐6‐bound beads (lane 4; Fig. S1D). These results suggested that the *Pf*PFD‐6 pulled down MSP‐1 from schizont stages.

### Homology modeling and docking of *Pf*PFD‐6 and MSP‐1

MSP‐1 is a multidomain protein comprising 235 kDa fam domain, MSP‐1C superfamily domain, and EGF domain (Fig. 4B). The three‐dimensional (3D) models for *Pf*PFD‐6 and *Pf*MSP‐1 were generated by I‐TASSER, and protein–protein docking studies were performed to analyze the interaction interface [53]. The generated models were subjected to refinement using 3D refine [54] and were verified using Procheck and ERRAT web servers to check the stereochemical quality and overall quality of predicted models [56, 57]. We found that 3D models of *Pf*PFD‐6 and MSP‐1 were sufficiently robust for protein docking studies. Ramachandran plots of the modeled structures generated by PROCHECK showed 99.9% and 89% residues to lie in the core region for *Pf*PFD‐6 and MSP‐1, respectively, while the overall quality factor obtained from the ERRAT score was 93% and 94%, respectively. Modeled structure of *Pf*PFD‐6 subunit comprises two long α‐helices connected by a short β‐hairpin (Fig. 4C‐i) and depict similar architecture to its homologs in archaea and humans [23, 28]. 3D structure of MSP‐1 showed helical structure linked with disordered loops (Fig. 4C‐ii).

We observed docking energy for *Pf*PFD‐6‐MSP‐1 complex as −234.96 kJ·mol^−1^ that indicates the stability of the docked complex (Fig. 4C‐iii). To identify key residues involved in interactions, the residue interaction network (RIN) profiles of docked complex were generated using RING 2.0 web server [58]. Analysis of docked structure and RIN plot showed that distal end of *Pf*PFD‐6 tentacles is involved in binding with MSP‐1. We observed that Asp104 and His108 of *Pf*PFD‐6 form maximum number of interactions with MSP‐1 (Fig. 4D).

## Discussion

Prefoldins facilitate folding of nascent polypeptide chains mainly actin and tubulin in eukaryotes and archaea, and also perform nuclear functions in yeast, plants, and *C. elegans* [29, 36, 37, 38, 39, 40]. PFD is a heterohexameric protein consisting of two α subunits and four β subunits, showing a jelly fish‐like appearance [23, 28]. Sequence analysis reveals that the presence of either α‐ or β‐Prefoldin domain is the characteristic feature of PFD subunits [23]. Although PFDs are well‐studied in several organisms, the functions of PFDs in malaria parasite are largely unknown. Transcriptome data from PlasmoDB show the existence of PFDs (PF3D7_1107500, PF3D7_1416900, PF3D7_0718500, PF3D7_0904500, PF3D7_1128100, PF3D7_0512000, and PF3D7_0907300) in the *Plasmodium* parasites. Despite the presence of a common domain (α‐ or β‐Prefoldin domain) with the same average length (~ 120 and ~ 140 amino acids for β‐subunits and α‐subunits, respectively), sequence polymorphism exists in the *Pf*PFD subunits. In the present study, we have chosen *Pf*PFD‐6 for the functional characterization and attempted to assign a possible role to it.

We have cloned full‐length *Pf*PFD‐6 gene in pET28a and pGEX‐4T1 vector. Expression of full‐length construct was scaled up in *E. coli* expression system BL21 (DE3) to purify protein from the soluble fraction using various chromatography techniques. Stage‐specific expression of the *Pf*PFD‐6 subunit from RT–PCR revealed its expression at all three asexual blood stages of parasites. *In vivo* expression of *Pf*PFD‐6 subunit is clearly evident from our western blot analysis on schizont stage parasite lysates, which shows a single band at the expected size for *Pf*PFD‐6 (13 kDa). Our IFA data demonstrate that the expression of *Pf*PFD‐6 subunit began from trophozoite stage and continued at schizont stage of asexual parasite life cycle. Yeast two‐hybrid data from Plasmodb suggested MSP‐1 as the interacting partner of *Pf*PFD‐6. Therefore, we perform colocalization experiments with anti‐*Pf*PFD‐6 and anti‐MSP‐1 antibodies. The *Pf*PFD‐6 was observed to have negligible colocalization with the merozoite surface protein‐1 (MSP‐1) at the mature trophozoite stages of parasite development. At later stages, both proteins showed maximum colocalization. Localization signals of MSP‐1 observed in our IFA are coherent with a previous report that suggests that MSP‐1 is synthesized at the mid‐trophozoite at asexual blood stage and transported to the parasite's plasma membrane [61].

Further, we tested the binding of *Pf*PFD‐6 with MSP‐1 by using GST pull‐down assays. Our binding assays revealed that *Pf*PFD‐6 is able to pull down MSP‐1 from parasite lysate (Fig. 4A). Therefore, it clearly illustrate for the first time that *Pf*PFD‐6 interact with MSP‐1. *P. falciparum* MSP‐1 has been studied extensively as a vaccine candidate antigen [62] and is reported to be essential for parasite viability [11]. Merozoite surface location of MSP‐1 has implicated its role in erythrocyte invasion. A report by Blackman *et al*. [63] suggested that antibodies against MSP‐1 inhibit erythrocyte invasion. MSP‐1 is also known to bind with erythrocyte glycophorin A [64] Band 3 [65, 66], and heparin‐like molecules [67, 68]. At mid‐trophozoite stage, MSP‐1 is primarily synthesized as a large molecular size precursor of ~ 200 kDa [61] and is proteolytically processed into four fragments, that is, 83 kDa, 30 kDa, 38 kDa, and C‐terminal 42 kDa (MSP1‐42) just before egress from the schizonts [12, 13, 14, 15, 16]. Following cleavage, MSP‐1 fragments remain noncovalently attached to the merozoite surface until invasion. The noncovalent MSP‐1 complex polypeptide fragments shed from the merozoite surface following proteolysis, and only a small C‐terminal fragment is carried into the erythrocyte. From our interaction studies, we propose that *Pf*PFD‐6 may play a possible role in MSP‐1 stability and trafficking, and have an indirect role in host cell invasion. However, this function needs to be confirmed *in vivo*.

We performed *in silico* analysis to investigate interaction between *Pf*PFD‐6 and MSP‐1. To date, there is no experimentally solved structure for *Plasmodium* chaperone. In this study, we attempted for the first time to predict the reliable model structure of *Pf*PFD‐6 subunit and performed docking with modeled structure of MSP‐1. RING web server was used to explore crucial residues of *Pf*PFD‐6 interacting with MSP‐1. Analysis of docked structure and RIN plot revealed that distal end of tentacles of *Pf*PFD‐6 bind to MSP‐1. A previous report has also suggested that distal tentacle end is required for interaction with non‐native substrate, while β‐hairpin of PFD subunits is involved in oligomerization [29, 34, 69].

Overall, our studies highlight information on an unexplored *Pf*PFD member and are likely to shed new light on malaria biology. Our studies using *in vivo*, *in vitro*, and computational approach form the outline for understanding *Pf*PFD‐6 expression during asexual blood stages of parasite and its interaction with MSP‐1. Further *in vivo* experiments would help in getting better insight into the role of *Pf*PFD‐6 in malaria biology.

## Conflict of interest

The authors declare no conflict of interest.

## Author contributions

VK conducted experiments, analyzed data, and wrote the manuscript. RS conducted experiments and helped in manuscript writing. AB conducted bioinformatic work, and wrote and edited the manuscript. AM conducted some cloning and pull‐down experiments. MA edited the manuscript. SS conceived the idea, analyzed the data, wrote the manuscript and approved the final draft.

## Supporting information


**Fig. S1.** Expression profile of *Pf*PFD‐6 gene at asexual blood stages, determination of antibody specificity and pull‐down assay of *Pf*PFD‐6 with MSP‐1.

## Data Availability

The datasets generated and/or analyzed during the study are included within the article and are available from the corresponding authors on reasonable request.
