# Supplementary material for: Prefoldin subunit 6 of Plasmodium falciparum binds merozoite surface protein‐1
Source: FEBS Open Bio. 2022 Mar 29;12(5):1050–60. doi: 10.1002/2211-5463.13022 (PMC9063436; doi:10.1002/2211-5463.13022)
Supplement: Supplementary file 1 — Fig. S1. Expression profile of PfPFD‐6 gene at asexual blood stages, determination of antibody specificity and pull‐down assay of PfPFD‐6 with MSP‐1. [file FEB4-12-1050-s001.docx]

**
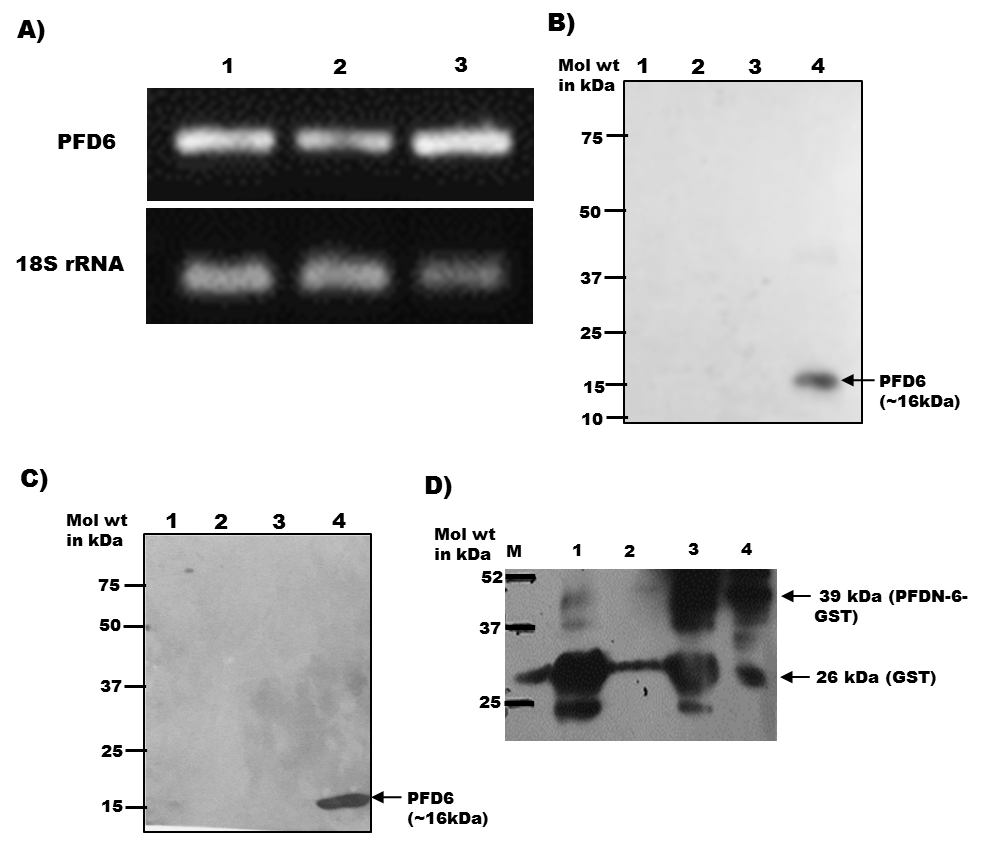
**

**Fig S1:** *Expression profile of PfPFD-6 gene using RT PCR and determination of antibody specificity*. (A) Expression profile of PFD-6 gene was checked at three different asexual stages of parasite life cycle using specific *Pf*PFD-6 primers (upper panel). 18s primer was used as a loading control (lower panel) Lane 1: ring stage (16-20 hrs), lane 2: trophzoite stage (32-38 hrs) and lane 3: schizont stage (40-46 hrs). (B) Western blot showing antibody specificity for recombinant purified *Pf*PFD-6 probed with rabbit anti-PFD6 antisera. Lane 1: BL 21 (DE3) *E. coli* lysate, lane 2: uninfected RBC pellet, lane 3: uninfected RBC cytosol and lane 4: *E. coli* BL21 (DE3) lysate containing recombinant plasmid for *Pf*PFD-6. (C) Western blot showing antibody specificity for recombinant purified *Pf*PFD-6 probed with mice anti-PFD6 antisera. Lane 1: BL 21 (DE3) *E. coli* lysate, lane 2: uninfected RBC pellet, lane 3: uninfected RBC cytosol and lane 4: *E. coli* BL21 (DE3) lysate containing recombinant plasmid for *Pf*PFD-6. (D) Pull down assay of *Pf*PFD-6 with MSP-1 probed with monoclonal anti-GST and polyclonal anti-*Pf*PFD-6 antibodies. Lane M: Protein ladder, lane 1: eluted fraction of GST bound beads, lane 2: supernatant of boiled GST bound beads, lane 3: eluted fraction of GST tagged *Pf*PFD-6 bound beads and lane 4: supernatant of boiled GST tagged *Pf*PFD-6 bound beads.
